# Computational Identification of Functional Centers in Complex Proteins: A Step-by-Step Guide With Examples

**DOI:** 10.3389/fbinf.2021.652286

**Published:** 2021-03-25

**Authors:** Wei Zhou, Wei Chi, Wanting Shen, Wanying Dou, Junyi Wang, Xuechen Tian, Christoph Gehring, Aloysius Wong

**Affiliations:** ^1^Department of Biology, College of Science and Technology, Wenzhou-Kean University, Wenzhou, China; ^2^Department of Computer Science, College of Science and Technology, Wenzhou-Kean University, Wenzhou, China; ^3^Department of Chemistry, Biology and Biotechnology, University of Perugia, Perugia, Italy; ^4^Zhejiang Bioinformatics International Science and Technology Cooperation Center of Wenzhou-Kean University, Wenzhou, China

**Keywords:** functional centers, moonlighting proteins, hidden domains, guanylate cyclase, adenylyl cyclase, nitric oxide sensors, abscisic acid receptor, H-NOX

## Abstract

In proteins, functional centers consist of the key amino acids required to perform molecular functions such as catalysis, ligand-binding, hormone- and gas-sensing. These centers are often embedded within complex multi-domain proteins and can perform important cellular signaling functions that enable fine-tuning of temporal and spatial regulation of signaling molecules and networks. To discover hidden functional centers, we have developed a protocol that consists of the following sequential steps. The first is the assembly of a search motif based on the key amino acids in the functional center followed by querying proteomes of interest with the assembled motif. The second consists of a structural assessment of proteins that harbor the motif. This approach, that relies on the application of computational tools for the analysis of data in public repositories and the biological interpretation of the search results, has to-date uncovered several novel functional centers in complex proteins. Here, we use recent examples to describe a step-by-step guide that details the workflow of this approach and supplement with notes, recommendations and cautions to make this protocol robust and widely applicable for the discovery of hidden functional centers.

## Key Points

- Functional centers have key roles in catalysis, ligand-binding, hormone- and gas-sensing, and are difficult to identify through standard homology approaches.
- Functional centers are often hidden in complex multi-domain proteins where they perform important molecular and cellular functions thereby enabling temporal and spatial modulation of signaling molecules and networks.
- Here we present a method for the detection of functional centers and provide a step-by-step guide that systematically describes the workflow and the computational tools employed in this protocol.
- This protocol also provides informative notes, recommendations and cautions at the appropriate steps to allow for a broad and robust application.

## Introduction

Functional centers are at the core of domains that contain the key amino acids required to perform a molecular function including, but not limited to, catalysis, ligand-binding, hormone- and gas-sensing (Wong et al., 2018). They are often embedded within complex multi-domain proteins and offer cryptic but important cellular signaling functions that afford intricate temporal and spatial regulation of signaling molecules and their signaling networks (Brady et al., 2007; Levskaya et al., 2009; Irving et al., 2012; Zhang and Ma, 2012; Freihat et al., 2014; Muleya et al., 2014). The lack of overall conservation of functional centers and the fact that they are often only a small part of complex proteins, have left them undetected by standard homology searches (Ludidi and Gehring, 2003; Guo and Fang, 2014; Wong et al., 2018). For instance, in higher plants, enzymes that generate and degrade cyclic mononucleotides or heme-based gas-sensing proteins were not identified until recently although their corresponding homologs exist in diverse groups of organisms ranging from bacteria to animals and humans (Martinez-Atienza et al., 2007; Domingos et al., 2015; Gehring and Turek, 2017; Swiezawska et al., 2018; Wong et al., 2020; Swiezawska-Boniecka et al., 2021; Turek and Irving, 2021). This is perplexing since the products and biological effects of the functional centers such as cyclic nucleotides-mediated cellular events (Bowler et al., 1994; Neuhaus et al., 1997; Durner et al., 1998; Maathuis and Sanders, 2001; Donaldson et al., 2004; Ederli et al., 2009; Isner and Maathuis, 2011; Pasqualini et al., 2011; Isner et al., 2012; Hartwig et al., 2014; Hussain et al., 2016; Marondedze et al., 2016) and nitric oxide mediated pollen tube chemotropic responses (Feijo et al., 2004; Prado et al., 2004, 2008; McInnis et al., 2006; Wang et al., 2009, 2012; Pasqualini et al., 2011, 2015; Domingos et al., 2015; Wong et al., 2020, 2021) have long been documented. Lately, a fundamentally different approach to the discovery of these molecular functions has been employed and has identified novel signaling components in complex proteins (Wong and Gehring, 2013b; Wong et al., 2015, 2018).

Based on the assumption that in complex multi-domain proteins only amino acids that directly perform a molecular function are conserved in the function centers, consensus sequence motifs that include only these key conserved amino acid residues, have been constructed and applied (Ludidi and Gehring, 2003; Gehring, 2010; Wong and Gehring, 2013a). These motifs are constructed by alignments of annotated functional centers from different and distantly related species and can then be used to query target proteomes—e.g., a model plant like *Arabidopsis thaliana*—to retrieve candidate proteins. The candidate proteins can subsequently be further assessed using homology modeling and molecular docking simulations prior to experimental testing. Functional annotation can also be done through the construction of sequence profiles on PROSITE which is a database of protein domains, families, and functional sites (Sigrist et al., 2013). These regions are better conserved throughout evolution especially in proteins from the same families and have largely similar three-dimensional structures which are crucial for a common molecular function (Wu et al., 2003; Lee et al., 2007; Marchler-Bauer et al., 2011; Mahlich et al., 2018). Functional centers deriving from annotated domains are either less obvious or have evolved beyond recognition in complex proteins because they often occupy a small part (<5%) of the entire protein and only harbor amino acids that are critical for functionality, which may explain why they were unaccounted for during *de novo* and homology sequence annotation. As they get incorporated into complex proteins of varying primary domains, their tertiary structures may also differ (Jeffery, 1999; Irving et al., 2018; Turek and Irving, 2021) (see Conclusion and Future Perspective for a specific example).

The sequential use of a motif search and structural assessment can overcome the challenges associated with identifying functional centers in complex proteins and indeed, several been identified in recent years as emerging evidence also suggests that many more await discovery (Turek and Gehring, 2016; Ooi et al., 2017; Wheeler et al., 2017; Al-Younis et al., 2018; Chatukuta et al., 2018; Bianchet et al., 2019; Freihat et al., 2019; Ruzvidzo et al., 2019; Wong et al., 2020). Given the highly varied nature of the functional centers, an automated pipeline is currently not feasible for all applications. Here, we use recent examples to document a step-by-step guide of the workflow and supplement it with detailed application notes, recommendations, and cautions. This will enable users to seamlessly apply this approach to the discovery of novel hidden functional centers in reference proteomes.

## Materials and Methods

### Required Software

In this protocol, the computer program required to generate the protein 3D models by homology modeling, is MODELLER (Sali and Blundell, 1993) which is available for download at https://salilab.org/modeller/download_installation.html. Users are required to register with a valid institutional email at https://salilab.org/modeller/registration.html prior to installation. To simulate docking of small molecules to the functional centers of the 3D models, AutoDock Vina (Trott and Olson, 2010) is used. This open-source program is available for download at http://vina.scripps.edu/download.html. AutoDockTools (ADT) (Morris et al., 2009) is a required graphical front-end for setting up and running Vina and can be downloaded as a package known as MGLTools at http://mgltools.scripps.edu/downloads. The package includes structure analysis and visualization programs such as Python Molecular Viewer (PMV) (Sanner, 1999). Alternatively, UCSF Chimera (Pettersen et al., 2004), which is available for download at https://www.cgl.ucsf.edu/chimera/download.html, can also be used to visualize protein structures and docking outcomes.

### General Workflow

**Figure 1:**
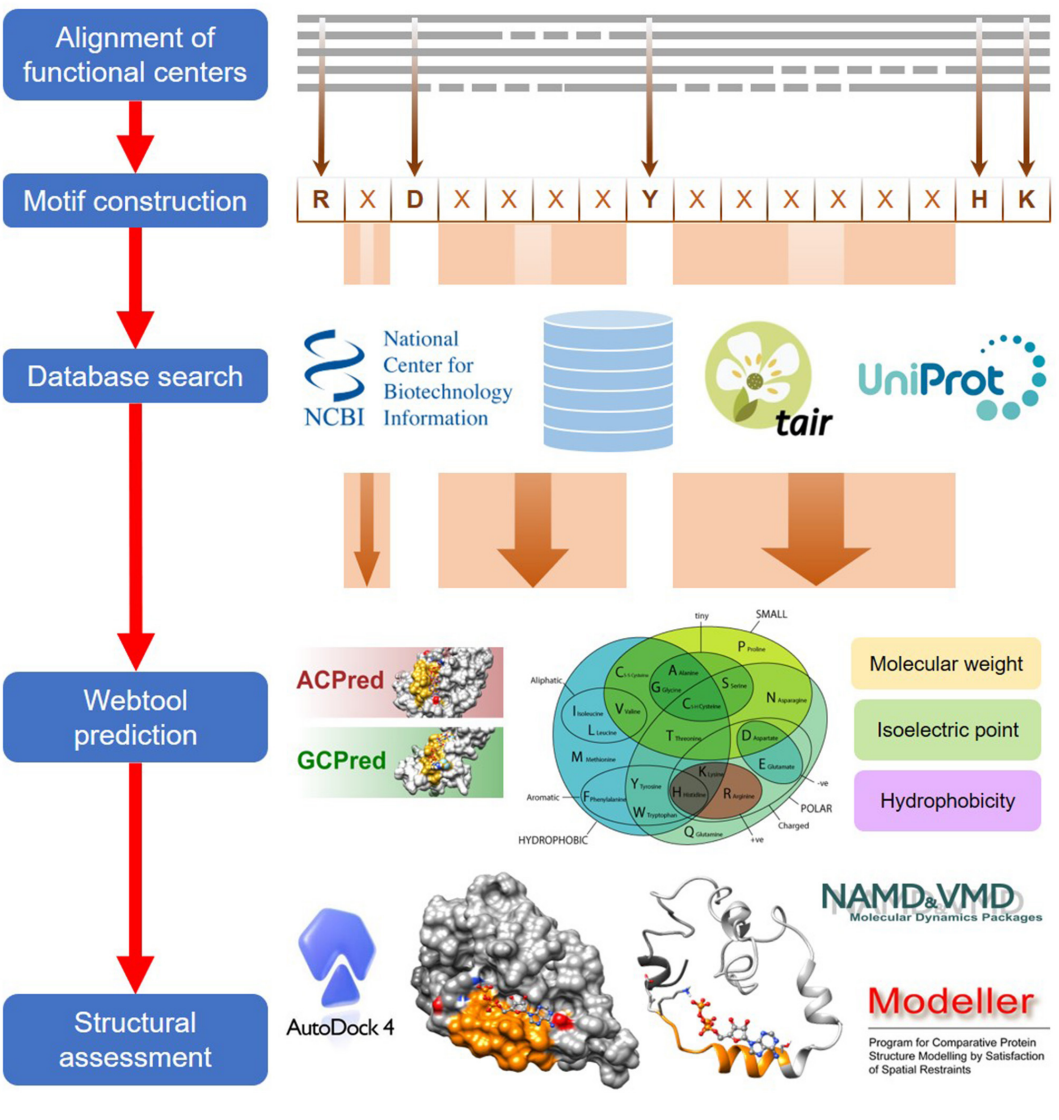
A general workflow for the computational identification of functional centers in complex proteins. The approach begins with an alignment of functional centers from proteins across species and followed by the construction of a consensus sequence that includes only conserved key amino acids which are separated by gaps as determined from the alignment. The consensus sequence now serves as a search motif to be queried on various databases to identify candidate functional centers and after which, they are screened on webtools if available, to assign confidence levels to the retrieved candidates. In the final step, top candidates are subjected to structural assessments that include model generations and docking simulations.

The computational approach to identify functional centers in complex proteins includes five general steps which are detailed in this section (Figure 1).

#### Step I: Alignment of Functional Centers

The first step of this approach involves the alignment of functional centers from a wide range of distantly related organisms. NOTE: The functional centers do not have to come from orthologs, and it is important to include sequences of experimentally validated proteins preferably from both prokaryotes and eukaryotes. This will increase the confidence of prediction by the amino acid motif constructed in the next phase. Functional centers may include catalytic sites, ligand- and hormone-binding sites or gas-sensing regions, as long as they are annotated as having a specific molecular function. NOTE: Only regions that directly participate in the molecular function should be considered as this protocol assumes a minimalistic strategy for functionality in highly diverse protein architectures. CAUTION: Functional centers typically range from 12 to 50 amino acids; an overly long sequence may reduce the chances of identifying promising candidates while a shorter sequence increases the chances of false positives.

#### Step II: Motif Construction

From the aligned sequences, highly conserved amino acids at each position in the alignment are included in a consensus sequence for the particular functional center. Amino acids that have been experimentally proven to perform key functions such as direct binding to ligands or essential for preserving certain charge and spatial configurations at the centers, are also included. These amino acids are indicated in square brackets []. NOTE: Key amino acids performing crucial molecular functions are also normally highly conserved at the functional centers of non-orthologous sequences and it is not uncommon for two or three amino acids with similar physicochemical properties to occupy a position. RECOMMENDATION: To be inclusive, we recommend including amino acids with similar chemical properties in the consensus sequence e.g., [RK] or [DE]. Detailed description of motif construction and its application have been described in Wong and Gehring (2013a,b), Wong et al. (2015, 2018).

Amino acids between the conserved residues and whose biochemical functions are unknown, may be excluded from the consensus sequence. They are indicated as “X” which stands for any of the 20 amino acids. From the aligned sequences, “X” may be several amino acids long and could assume differing ranges in centers from different species or non-orthologous sequences and the gap size is noted in brackets e.g., (N,M) or {N,M}, with “N” and “M” representing the minimum and maximum number of amino acids. CAUTION: Consult the pattern syntax of the respective tools to avoid errors.

After evaluating the conserved and non-conserved amino acids at each position of the alignment, the consensus sequence for the particular functional center is ready for application. CAUTION: Check the consensus sequence for errors and inconsistencies, e.g., odd or contrasting amino acid properties, at each position. RECOMMENDATION: A 12–50 amino acid long consensus sequence is typically a good starting point and motifs can be modified to increase or loosen stringency. NOTE: Shorter sequences can be considered if there are many specifically conserved residues and longer sequences can be considered if the conserved residues are too few and far apart. For a specific example of motif construction, please refer to the “Identification of heme-containing gas sensors in complex proteins” section in Results and Discussion and the sequence alignment of H-NOX domains in Supplementary Figure 1 or contact the corresponding author directly to obtain the most up to date datasets and output files.

#### Step III: Database Query

The consensus sequence now serves as a search motif to query any protein databases for candidate proteins harboring given functional centers. RECOMMENDATION: We recommend the use of the ScanProsite tool (Gattiker et al., 2002) available at: https://prosite.expasy.org/scanprosite that allows a search against protein database for known motifs (option 1) or with custom motifs (option 2). For proteins from *Arabidopsis thaliana*, the PatMatch tool (Yan et al., 2005) available at: https://www.arabidopsis.org/cgi-bin/patmatch/nph-patmatch.pl can be used. CAUTION: Consult the pattern syntax of the respective tools to avoid errors. NOTE: If the retrieved candidates are few, the consensus sequence (see Step II) can be revisited to relax the stringency of the motif by varying the gap sizes between conserved amino acids and/or by adding amino acids with similar chemical and physical properties to the conserved positions (e.g., instead of [IL] expand to [VIL]). CAUTION: A long list of candidates may indicate high false positive rates but at this point, this is not a concern because the retrieved hits can be conveniently screened and filtered with various webtools that provide statistics for confidence levels and candidate rankings in the next step. The motif only needs to be revised to increase stringency if no such webtools are currently available.

#### Step IV: Webtool Screening and Filtering

Candidates retrieved from the motif search can be screened with predictive tools for the particular molecular function if such tools are available. These webtools consider the physical and chemical properties of non-conserved amino acids in experimentally validated functional centers in addition to their conserved residues where their algorithms generate statistics that compare the queried sequence to the mean values of the experimentally validated pool of proteins (Xu et al., 2018a,b). Users can therefore use these statistics to rank retrieved hits from high to low confidence levels. RECOMMENDATION: We recommend the use of these webtools not only to increase confidence in the retrieved candidates but also to enable high-throughput processing of a long list of candidates. CAUTION: Although these webtools are designed to predict hits with high confidence, they remain predictive in nature and thus may not detect some positive hits especially in poorly characterized proteins. RECOMMENDATION: If the number of retrieved candidates is small and there are biochemical and cellular justifications supporting the functions of those hits, we recommend users to proceed with the next step involving structural assessment even if the webtool screening returns no hits. NOTE: At this point, it may be advantageous to inspect the candidate list and sort them into gene ontology (GO) categories to help selection of proteins with functions of particular interest before proceeding to the structural examination.

#### Step V: Structural Assessment

Candidates will now be assessed structurally and since this is the most computationally intense part and requires the most interpretation, the list of candidates should ideally be small. RECOMMENDATION: For a longer list of candidates, the top 5–10 proteins determined with the webtool (Step IV) can be selected for structural assessments. The assessment includes the generation of three-dimensional (3D) structures in case there are no known crystal structures for the candidates and conducting molecular docking simulations to predict functionality. NOTE: This phase requires installation of several software such as MODELLER, AutoDock Vina, MGLTools and UCSF Chimera, and a level of familiarity with these software. RECOMMENDATION: Users are recommended first to familiarize themselves with the operations of the required software packages before attempting this step, as this is a crucial phase for predicting functionality.

3D models should first be generated for candidates that have no known crystal structures by homology modeling using MODELLER (Sali and Blundell, 1993). This software generates protein 3D structures from amino acid sequences based on spatial restraints in template structures provided by the user. NOTE: See required software section for installation and registration guide. The five steps in homology modeling by MODELLER are: selection of crystal structures related to the candidate, template structure selection, alignment of candidate amino acid sequence to the template, model generation, and model evaluations. A detailed tutorial, the required program files, example input and output files in.zip format (for Windows) or.tar.gz format (for Unix/Linux), and the relevant scripts to run MODELLER can be found at: https://salilab.org/modeller/tutorial/basic.html. NOTE: These are the complete steps for homology modeling, but users can choose to perform alignments and template selection with other programs such as BLASTp (McGinnis and Madden, 2004) available at: https://blast.ncbi.nlm.nih.gov/Blast.cgi?PAGE=Proteins. If using BLASTp, the “Protein Data Bank” database should be selected to only retrieve results with known crystal structures. CAUTION: Selection of template structure is a critical step in determining the quality of the model. If using BLASTp, a crystal structure with high identity with the queried candidate in the particular region where the functional center resides will be more suitable than another structure with higher overall coverage and/or max. score but lower identity at the corresponding functional center region. This is particularly relevant since moonlighting functional centers constitute only a small part of complex multi-domain proteins (Su et al., 2019).

Once a high-quality 3D model is obtained, molecular docking simulation can be performed using AutoDock Vina (Trott and Olson, 2010) which is an automated docking software for the prediction of the binding ligands to a receptor protein. NOTE: See required software for download and installation guides. CAUTION: Prior to running AutoDock Vina, necessary files must be prepared using AutoDockTools (ADT) (Morris et al., 2009) which is a graphical front-end software for setting up and running AutoDock. RECOMMENDATION: We recommend users to download the entire MGLTools package which include ADT and Python Molecular Viewer (PMV) (Sanner, 1999) for visualization of protein structures. Alternatively, users can also install UCSF Chimera (Pettersen et al., 2004) which is a tool for interactive visualization and analysis of molecular structures. All software downloads and installations are detailed in the software description. A detailed step-by-step tutorial on “.pdbqt” file preparation with ADT, example input and output files in.zip format, and running docking simulations using Vina and PMV is available at: http://vina.scripps.edu/tutorial.html. CAUTION: When setting up the grid box, the position and size determination is critical for the success of molecular docking. An overly small or off-position grid box will result in failure to identify suitable binding poses during docking simulation. The grid box volume should not only cover the entire area of the functional center but also be large enough to allow free rotation of the ligand at its most extended configuration.

After docking simulations with AudoDock Vina, binding poses can be evaluated on PMV or Chimera. By default, AutoDock Vina will rank binding poses based on free energies. NOTE: Binding poses of ligands at some functional centers are already known based on experimental data and, in such instances, these ligand orientations should be given priority over their free energy calculations. Further docking simulations can be conducted to evaluate the effect of mutations of key residues at the functional centers.

## Results and Discussion

Using the general workflow as a guide, we describe specific applications using recent examples to detail the step-by-step process that led of the identification of catalytic centers and gas-sensing and hormone-interacting sites in complex proteins.

### Identification of Catalytic Centers

**Figure 2:**
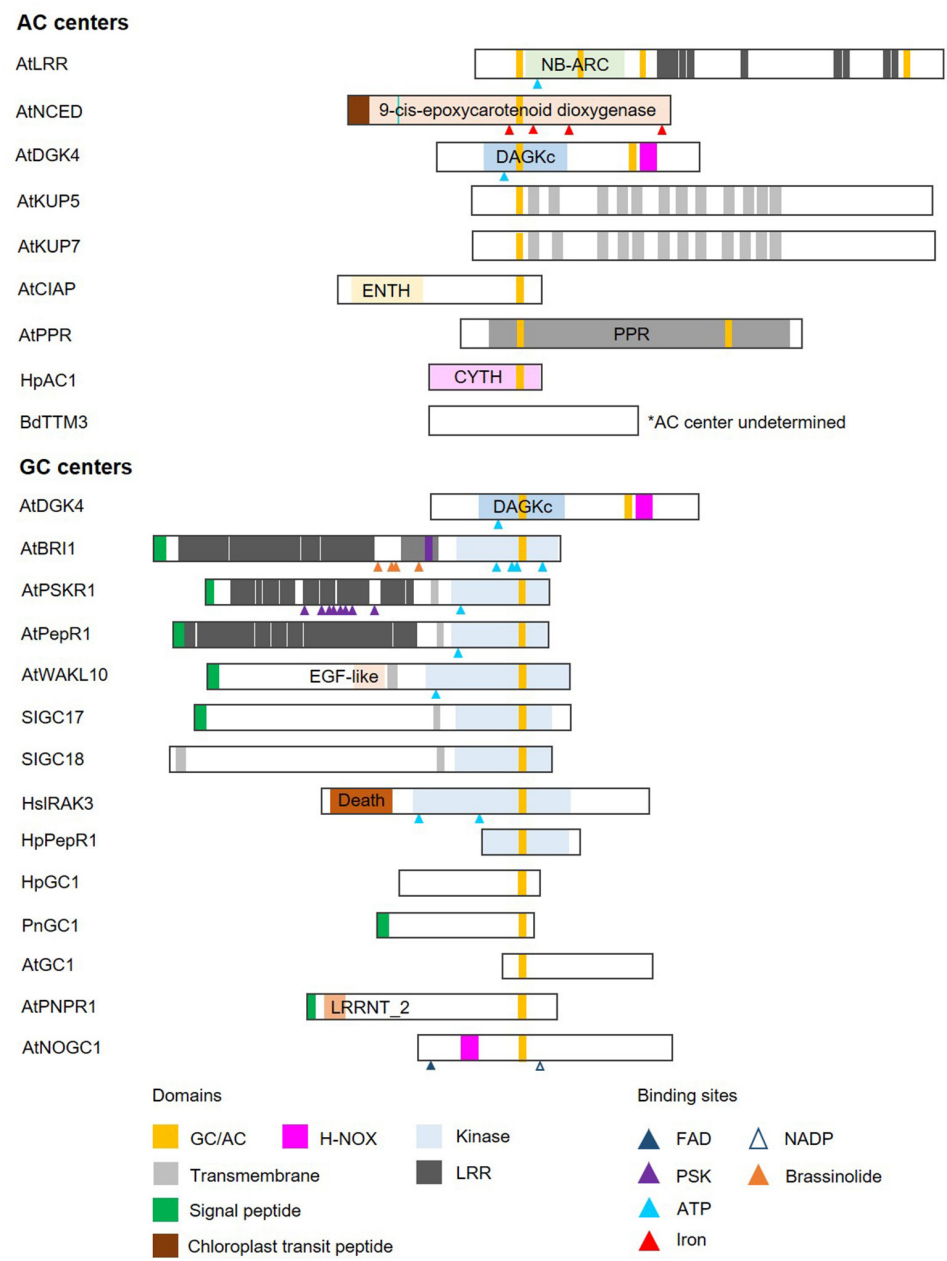
Domain architecture of proteins containing nucleotide cyclase functional centers. The domain organizations of experimentally validated GCs and ACs identified using a motif-based approach from *Arabidopsis thaliana* (At), *Brachypodium distachyon* (Bd), *Solanum lycopersicum* (Sl), *Hippeastrum* (Hp), *Pharbitis nil* (Pn), and *Homo sapiens* (Hs), are illustrated as 2-dimensional bars and aligned at their corresponding GC/AC domains. Protein UniProt IDs are as follows: AtLRR (Q9LRR5), AtNCED (Q9LRR7), AtDGK4 (Q1PDI2), AtKUP5 (Q8LPL8), AtKUP7 (Q9FY75), AtClAP (Q9C9X5), AtPPR (Q9SXD8), HpAC1 (E1AQY1), BdTTM3 (I1I2P2), AtBRI1 (O22476) (see Supplementary Figure 2 for an illustration of AtBRI1 protein topology), AtPSKR1 (Q9ZVR7), AtPepR1 (Q9SSL9), AtWAKL10 (Q8VYA3), SlGC17 (A0A3Q7FS62), SlGC18 (A0A3Q7FY08), HsIRAK3 (Q9Y616), HpPepR1 (A0A1U9X9S6), HpGC1 (D9MWM6), PnGC1 (Q0PY32), AtGC1 (Q8L870), AtPNPR1 (F4HR92), and AtNOGC1 (Q9SXD9). *AC center undetermined.

Cyclic mononucleotide cyclases (guanylate cyclase and adenylate cyclase; GC and AC) are enzymes that catalyze the conversion of GTP and ATP to their cyclic forms, cGMP, and cAMP, respectively. In line with the concept of functional centers (Wong et al., 2018), only catalytic centers of canonical GCs and ACs are conserved non-orthologous cyclases. Currently available experimental data indicate that these enzymatic centers are often part of complex proteins with other primary domains and highly varied domain architectures (Figure 2).

#### A Step-by-Step Guide for the Identification of Cyclic Mononucleotide Cyclases

**Figure 3:**
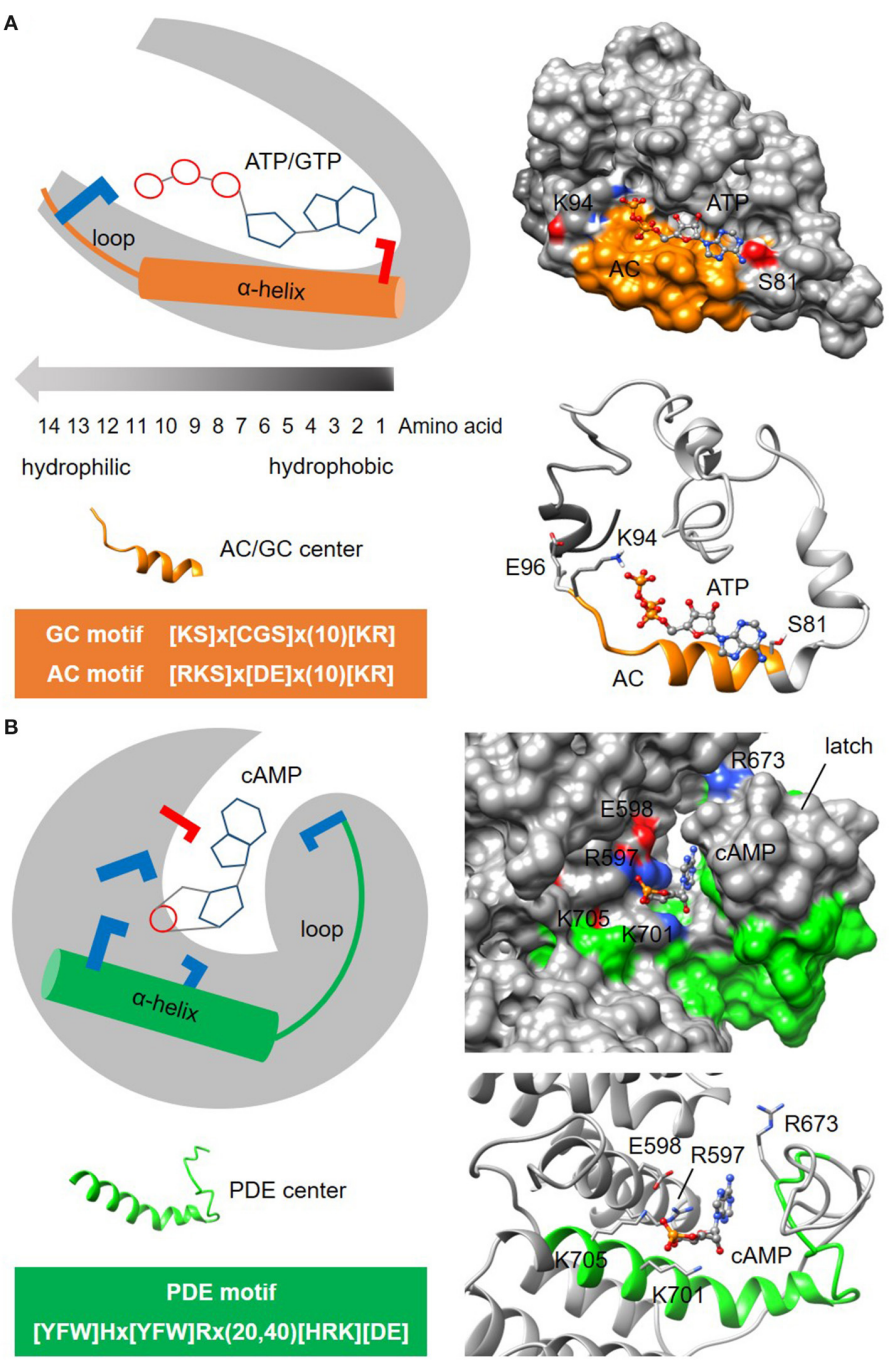
Representative structures of nucleotide cyclase and phosphodiesterase functional centers. **(A)** The typical nucleotide cyclase center identified through a 14-amino acid long search motif as exemplified by an adenylyl cyclase (AC) in an *Arabidopsis* potassium channel AtKUP5 (Al-Younis et al., 2018), assumes an alpha-helical secondary fold that is followed by a loop. At the tertiary level, the AC center typically forms a clear cavity that could dock with the substrate ATP in a binding pose where the adenine points into the cavity toward the amino acid at the first position of the motif, and the phosphate points outwards toward the positively charged [KR] amino acid at the solvent exposed region of the cavity. Negatively and positively charged amino acids that are crucial for the interactions with the substrate are colored red and blue, respectively. **(B)** The putative phosphodiesterase center in an *Arabidopsis* potassium channel AtKUP5 identified through a 27-47 amino acid long search motif, assumes an alpha-helical secondary fold that is followed by a loop which forms the latch region enclosing the docked cAMP substrate within a distinct cavity (Kwiatkowski et al., 2021). Negatively and positively charged amino acids crucial for the interactions with the substrate are colored red and blue, respectively.

1. Alignment of catalytic centers of GCs or ACs from organisms across species including prokaryotes and eukaryotes. NOTE: For a specific example of motif construction, please refer to the “Identification of heme-containing gas sensors in complex proteins” section in Results and Discussion and the sequence alignment of H-NOX domains in Supplementary Figure 1 or contact the corresponding author directly to obtain the most up to date datasets and output files.
2. Determining the consensus sequence of GC or AC catalytic centers by including only the conserved key amino acids and separated by rational gaps made up of non-conserved amino acids. NOTE: The current consensus sequences for GC and AC functional centers are 14-amino acids long with negatively charged amino acids D or E (written as [DE] in the motif to indicate that both amino acids can function in this position) 0–3 positions downstream of the centers (Figure 3A).
3. Querying protein databases with GC or AC motifs to identify candidates GCs and ACs. NOTE: The currently tested motifs for GC and AC functional centers are [KS]x[CGS]x(10)[KR] and [RKS]x[DE]x(10)[KR], respectively (Figure 3). CAUTION: Consult the pattern syntax of the respective tools to avoid errors. Derivatives of these motifs justified by experimental evidence or rational modifications have also been described in detail (Kwezi et al., 2007; Mulaudzi et al., 2011; Bianchet et al., 2019; Ruzvidzo et al., 2019).
4. Application of GCPred and/or ACPred webtools [available at: http://gcpred.com (Xu et al., 2018a) and http://gcpred.com/acpred (Xu et al., 2018b)] for the assessment of confidence levels of retrieved candidates. These webtools contain algorithms that compare the physicochemical values of amino acids such as their molecular weights, isoelectric points and hydrophobicity to mean values of experimentally validated functional centers. RECOMMENDATION: We propose selecting the “cation-binding” option because current experimental data indicate that GCs and ACs of such nature have higher activities when these negatively charged amino acids [DE] are present at 1, 2, or 3 amino acids downstream of the functional centers. NOTE: According to the interpretation guide (Xu et al., 2018b), a score of 0-1 for each physicochemical parameter is generated, where 1 is closest to the mean of experimentally validated GC or AC functional center. Green scores represent “high” confidence and red ones represent “low” confidence. We also recommend that the users select candidates with at least two green physicochemical values in addition to the compulsory green overall mean score. Good candidates should contain no red scores. CAUTION: Although these webtools are designed to predict hits with high confidence, they remain predictive in nature and thus may not detect some positive hits especially in lesser studied proteins. RECOMMENDATION: If the retrieved list of candidates is small and there are justifications, e.g., from the literature, supporting the plausibility of the functions, we recommend that users proceed with the next phase involving structural assessment.
5. Model generation and docking simulation with MODELLER and AutoDock Vina to evaluate the structures of individual candidates and to predict the binding of substrate i.e., GTP or ATP, since this is a prerequisite for catalysis. NOTE: Based on experimentally validated GC and AC centers, they contain a characteristic alpha-helix barrel followed by a loop secondary fold (Wong and Gehring, 2013b; Wong et al., 2015, 2018; Al-Younis et al., 2018) (Figure 3A). Furthermore, experimentally validated GC and AC centers have the following substrate binding pose: purine bases adenine or guanine facing into the cavity toward amino acid at the first position of the motif in the hydrophobic interior while the phosphate end facing the positively charged amino acid [KR] at the opening of the cavity (Al-Younis et al., 2018; Ruzvidzo et al., 2019). If structural evaluations determine that such substrate orientations are feasible in new candidates, then the confidence level is further increased.

In general, candidates retrieved from the motif search that rank highly in the webtool predictions and display the described structural features, will be considered suitable candidates for experimental verifications to ascertain their GC or AC activities both *in vitro* and *in vivo*.

Another example to illustrate the search for enzymatic functional centers are cyclic nucleotide phosphodiesterases (PDEs) which are enzymes that degrade the cyclic mononucleotides cGMP or cAMP. Much like nucleotide cyclase functional centers, PDEs may only retain the key conserved amino acids at the catalytic centers in complex multi-domain proteins and are therefore beyond the detection limit of BLAST searches. The steps involved in the identification of PDE centers follow the same guide as the cyclic mononucleotide cyclases. NOTE: Currently, the motif for PDE center is 27-47 amino acid long and is determined to be [YFW]Hx[YFW]Rx(20,40)[HRK][DE] (Figure 3B). CAUTION: The current PDE motif is stringent and identifies candidates with high confidence. As experimental evidence for PDE centers begin to emerge, the motif may eventually be relaxed to allow for the identification of novel candidates. In the absence of webtools for the prediction of PDE centers, retrieved candidates will be structurally evaluated in the next step. NOTE: Current model for PDE centers has a distinct cavity that accommodate cyclic mononucleotides within a latch. The PDE motif can form an alpha-helix secondary fold that is followed by a loop that makes up the latch (Kwiatkowski et al., 2021) (Figure 3B). Since PDE centers are less characterized than the centers of mononucleotide cyclases, the motif and structure presented here may yet undergo modifications that will improve prediction accuracy and coverage of hidden PDE centers.

### Identification of Heme-Containing Gas Sensors in Complex Proteins

**Figure 4:**
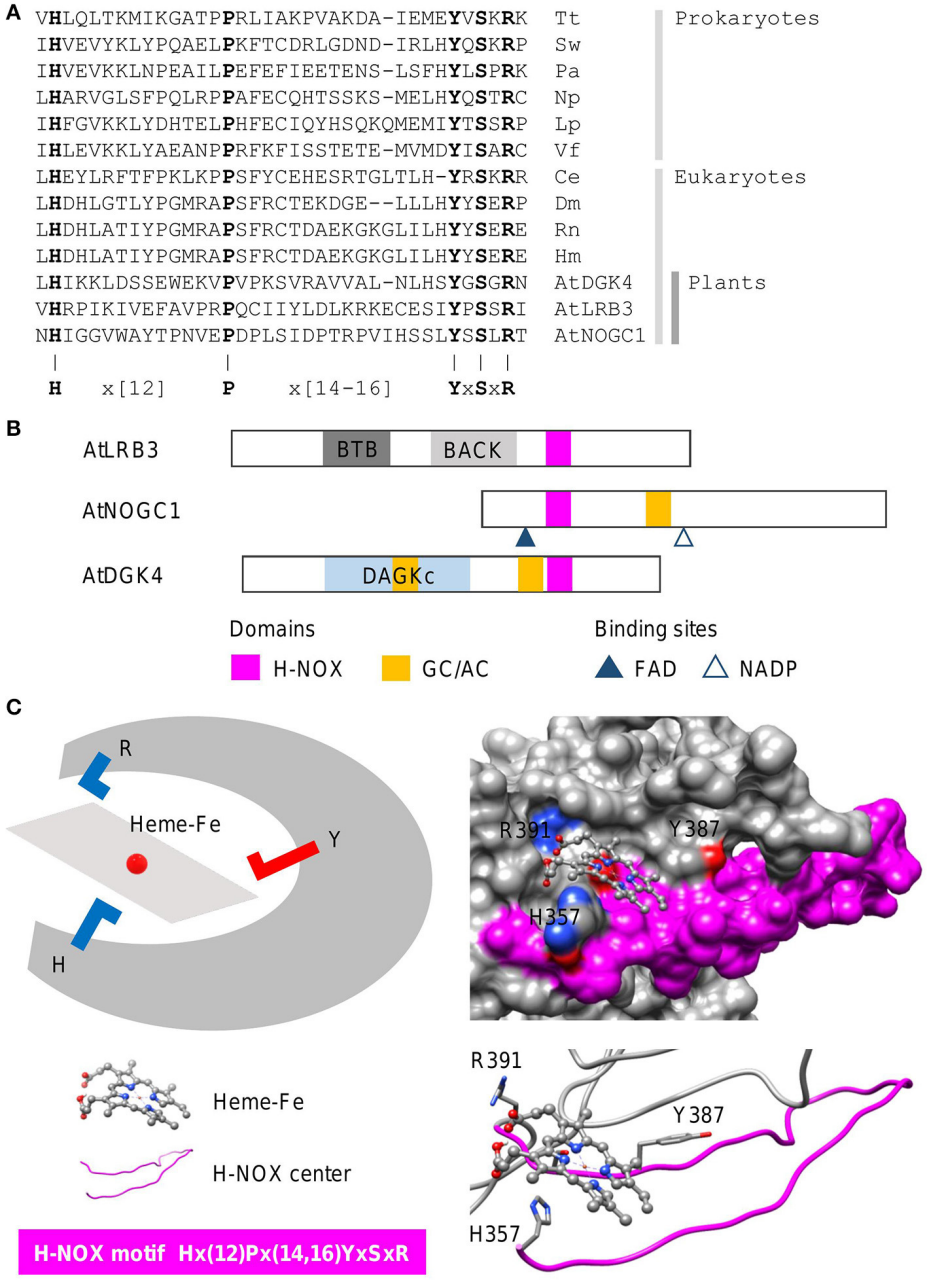
Sequence alignment and domain architecture of proteins containing H-NOX centers, and a representative structure of the H-NOX center. **(A)** Alignment of the heme-binding centers of H-NOX proteins from organisms across species. Tt, *Thermoanaerobacter tengcongensis* (UniProt ID: Q8RBX6); Sw, *Shewanella woodyi* (UniProt ID: B1KIH6); Pa, *Pseudoalteromonas atlantica* (UniProt ID: Q15VN4); Np, *Nostoc punctiforme* (UniProt ID: B2IZ76); Lp, *Legionella pneumophila* (UniProt ID: Q5WTZ5); Vf, *Vibrio fischeri* (UniProt ID: Q5E1F5); Ce, *Caenorhabditis elegans* (UniProt ID: Q86C56); Dm, *Drosophila melanogaster* (UniProt ID: Q24086); Rn, *Rattus norvegicus* (UniProt ID: P20595); Hs, *Homo sapiens* (UniProt ID: Q02153) and At, *Arabidopsis thaliana*. Bolded letters are conserved amino acids that are also experimentally shown to be crucial for heme-binding and stabilization. **(B)** The domain organizations of experimentally validated H-NOX centers identified using a motif-based approach from *Arabidopsis thaliana* (At), are illustrated as 2-dimensional bars, and aligned at their corresponding H-NOX centers (see Supplementary Figure 1 for full alignment of H-NOX domains). Protein UniProt IDs are as follows: AtLRB3 (O04615), AtDGK4 (Q1PDI2), and AtNOGC1 (Q9SXD9). **(C)** A representative structure of a protein containing the H-NOX center. The H-NOX center in an *Arabidopsis* BTB/POZ domain-containing protein AtLRB (Zarban et al., 2019), identified through a 33–35 amino acid long search motif, assumes a long loop that wraps the docked heme-Fe moiety within a clearly defined pocket. The “H” residue in the motif is the distal ligand that binds to the iron and the YxSxR signature stabilizes the heme through hydrogen bonding. Negatively and positively charged amino acids crucial for the interactions with the substrate are colored red and blue, respectively.

Heme-nitric oxide/oxygen (H-NOX) centers are heme-containing regions that can sense gases including the signaling molecule nitric oxide (NO). In line with the concept of functional centers (Wong et al., 2018), only amino acids at the H-NOX center occupied by the heme moiety, are conserved. The steps involved in the identification of heme-containing gas sensors follow the same guide as the cyclic mononucleotide cyclases. In this case, we show how an H-NOX consensus sequence can be constructed from the alignment of the heme-binding centers of H-NOX proteins from prokaryotes and eukaryotes. From the alignment, the “H” and “P” amino acids, as well as the “YxSxR” signature, are highly conserved across distantly related species (Figure 4A). Furthermore, these amino acids have been determined experimentally to have direct heme- and/or gas-binding functions at the H-NOX centers. For instance, the “H” residue in the motif serves as the distal ligand for NO (Olea et al., 2010) while the YxSxR signature stabilizes the porphyrin ring (Pellicena et al., 2004). Therefore, these amino acids are included in the consensus sequence with the non-conserved flanking amino acids that are not known to play functional roles at the H-NOX centers, assigned as “X” which stands for any of the 20 amino acids, and the gap size noted in brackets (N,M) or {N,M}, with “N” and “M” representing the minimum and maximum number of amino acids (Figure 4A). The constructed H-NOX consensus sequence was then used as a search term to identify heme-containing gas sensors in plants by querying the proteome of *Arabidopsis thaliana* and from which three candidates have since been experimentally confirmed to sense NO. NOTE: The current consensus sequence H-NOX center is 33–35 amino acids long and is determined to be Hx(12)Px(14,16)YxSxR (Figure 4). CAUTION: Consult the pattern syntax of the respective tools to avoid errors. Since there is currently no known webtools for the prediction of H-NOX centers, retrieved candidates will be directly subjected to structural evaluations in the next step. Current experimental data indicate that these H-NOX centers exist in complex proteins with other primary domains and varied architectures (Figure 4B). Mutations to either the “H” or “Y” residues impaired heme binding and concomitantly also the ability to sense NO (Figure 4C) (Zarban et al., 2019; Wong et al., 2020). In general, candidates retrieved from the motif search that display the described structural features, will be considered suitable candidates for experimental verifications to ascertain their gas-binding affinities both *in vitro* and *in vivo*. Currently, H-NOX motif is intentionally constructed to be rigid to identify candidates of high confidence especially in the absence of other predictive tools. Relaxation of the motif based on more data are likely to identify more heme-containing H-NOX proteins (Wong et al., 2021). RECOMMENDATION: We recommend omitting the “P” residue from the motif to increase coverage as this residue appears the least essential in terms of functionality.

### Identification of Hormone Binding Sites

**Figure 5:**
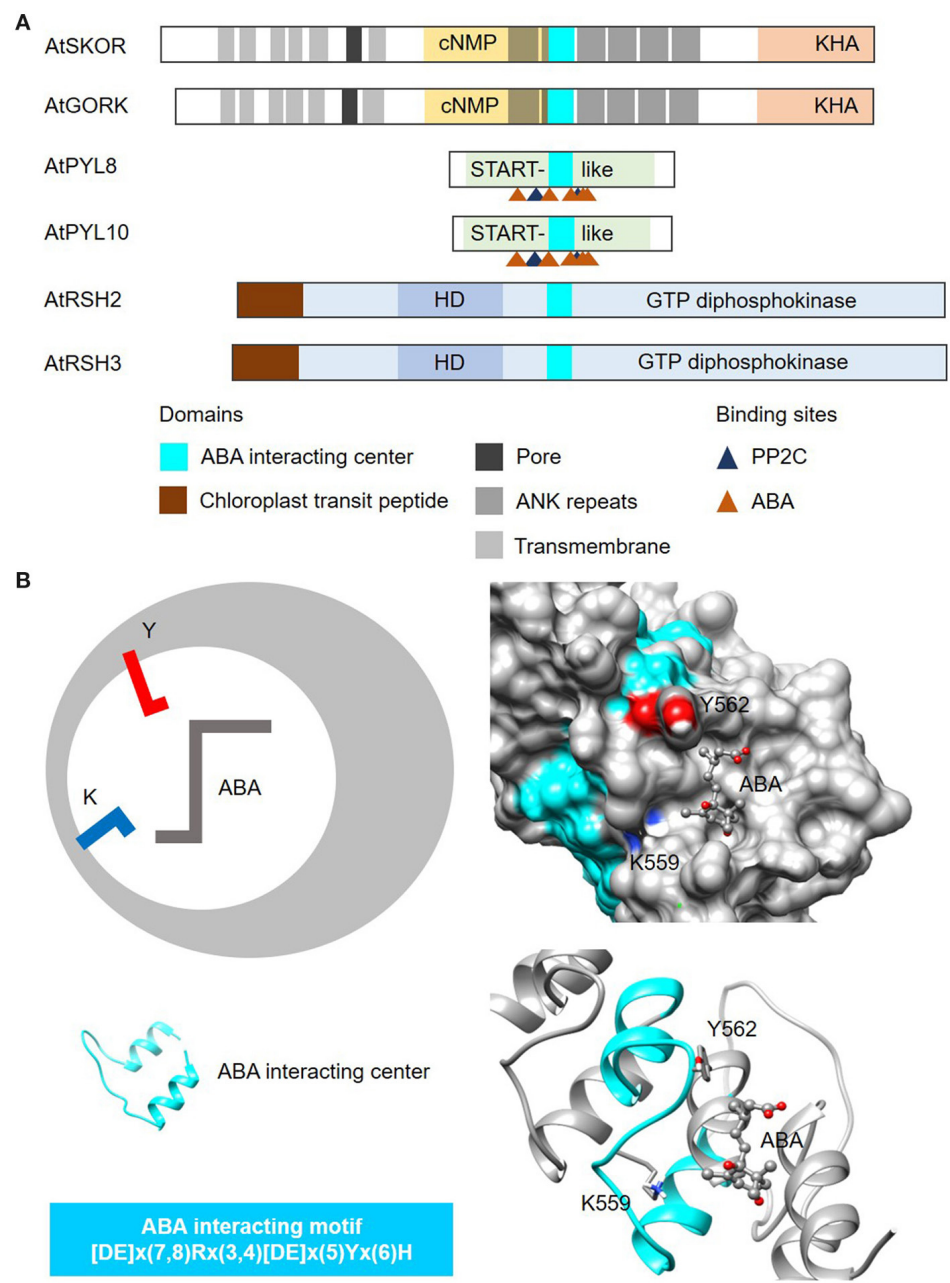
Domain architecture of proteins containing ABA-interacting centers and a representative structure of the ABA center. **(A)** The domain organizations of ABA-interacting centers identified using a motif-based approach from *Arabidopsis thaliana* (At), are illustrated as 2-dimensional bars and aligned at their corresponding H-NOX centers. Protein UniProt IDs are as follows: AtGORK (Q94A76), AtPYL8 (Q9FGM1), AtPYL10 (Q8H1R0), AtSKOR (Q9M8S6), AtRSH2 (Q9LVJ3) and AtRSH3 (Q9SYH1). AtGORK, AtPYL8 and AtPYL10 have been confirmed experimentally to be ABA receptors while AtSKOR, AtRSH2 and AtRSH3 harbor the ABA center motif and are known to response to ABA. **(B)** The ABA-interacting center in an *Arabidopsis* potassium transporter AtGORK (Ooi et al., 2017) identified through a 26-28 amino acid long search motif occupies a clear cavity that could dock with ABA with the “Y” and “K” residues being crucial for maintaining ABA affinity. Negatively and positively charged amino acids crucial for the interactions with the substrate are colored red and blue, respectively.

In this case, we use the amino acids directly involved in binding to the plant hormone abscisic acid (ABA) from the well-characterized START protein family PYR/PYLs (Melcher et al., 2009; Park et al., 2009), to identify other plant proteins that can bind to or interact with ABA (Figure 5A). The steps involved in the identification of ABA-interacting centers follow the same guide as the cyclic mononucleotide cyclases. NOTE: The current consensus sequence for the ABA-interacting center is 26–28 amino acids long and is determined to be [DE]x(7,8)Rx(3,4)[DE]x(5)Yx(6)H (Figure 5). CAUTION: Consult the pattern syntax of the respective tools to avoid errors. Since there is currently no known webtools for the prediction of ABA-interacting centers, retrieved candidates will be directly subjected to structural evaluations in the next step. NOTE: Based on experimentally validated proteins containing the ABA-interacting centers, the “Y” residue in the motif together with the “K” located three amino acids upstream, are crucial for ABA binding (Figure 5B). Mutations to either the “Y” or “K” residues impair ABA binding and concomitantly other functions of the candidate protein e.g., K^+^ transport (Ooi et al., 2017). Currently, the ABA-interacting motif is intentionally constructed to be relatively rigid to identify candidates of high confidence especially in the absence of predictive tools, but it can conceivably be made more relaxed to identify more ABA-interacting proteins. RECOMMENDATION: We recommend increasing the gaps upstream of the “Y” and the “H” residues in the motif to allow for a more inclusive prediction.

## Conclusion and Future Perspective

We have developed a general workflow and presented a step-by-step guide for the applications of this computational approach to identify functional centers in complex proteins using recent examples as case studies. While this protocol leverages on existing software and follows standard methods in protein functional annotations, the focus is however on the identification of functional centers that unlike canonical domains, consist of only the key amino acids required to perform certain molecular functions in complex proteins. Instead of detecting broadly the common patterns or motifs in proteins, we derived our motifs from key residues of functional centers in existing domains that have been experimentally proven to be fully functional.

The initial motivation was to identify the corresponding signaling components of animals and bacteria (Loewenstein et al., 2009) in plant systems many of which, are long taught to be absent because there were no orthologs in plants. It was hypothesized that these domains have become markedly altered to retain only their functional centers as they get incorporated into complex multi-domain proteins such as that in the well-characterized *Arabidopsis* BRASSINOSTEROID INSENSITIVE 1 (AtBRI1; UniProt ID: O22476). The 1,196 amino acid long AtBRI1 consists of an extracellular brassinosteroid receptor region, a transmembrane, and an intracellular kinase domain with the GC center embedded within the primary kinase domain. Both the hormone receptor region and kinase domain take up ~62.7 and 23.0% of the protein while the GC center makes up only 0.12% of the entire protein (Supplementary Figure 2). Due to the diverse architecture of complex proteins, this protocol identifies functional centers that are more targeted and may not necessarily have similar 3D structures as it would be with canonical domains. Their incorporation into multi-domain complex proteins of varying primary functions, make these functional centers “hidden” or undetectable, hence not annotated in protein domains and families of existing databases.

Our application-specific CAUTIONS, NOTES and RECOMMENDATIONS will enable users to seamlessly apply the out-lined protocol and thereby increase the chances of success in the discovery of hidden functional centers or ligands in complex proteins as it informs users of potential pitfalls and gives suggestions for optimization. For instance, the recent identifications of functional nucleotide cyclase functional centers in crop plants such as tomato (Rahman et al., 2020) and rice (Malukani et al., 2020), and in other economically important plants such as ornamentals *Hippeastrum sp*. (Swiezawska et al., 2015, 2017) and Japanese morning glory (Szmidt-Jaworska et al., 2009) and in the monocot grass model *Brachypodium* (Swiezawska et al., 2020), as well as in a human immune-responsive protein IRAK3 (Freihat et al., 2019), have demonstrated the robustness of this approach and its significance in biological discoveries across different systems. Furthermore, the method enables candidate selections for follow-up *in vivo* and *in planta* studies that will eventually reveal the biological roles of these functional centers such as those reported in Joudoi et al. (2013), Shen et al. (2019), Vaz Dias et al. (2019), Angkawijaya et al. (2020), Lee et al. (2020), and Turek et al. (2020). We foresee that emerging experimental data will inform and strengthen motif refinement efforts, and enable the development of modern machine learning techniques that incorporate multiple features ranging from the classical physicochemical properties of protein domains and protein-protein interaction (PPI) networks to GO based function predictions, to not only automate annotations for uncharacterized proteins, but also to identify hidden functional centers in complex multi-functional proteins (Rifaioglu et al., 2019; Bonetta and Valentino, 2020; Cai et al., 2020; Littmann et al., 2021).

## Data Availability Statement

The original contributions presented in the study are included in the article/Supplementary Material, further inquiries can be directed to the corresponding author/s.

## Author Contributions

AW conceived the project. WC, WZ, WS, WD, JW, and AW collected the data. AW, XT and CG analyzed the data. AW and CG wrote the manuscript. All authors contributed to the article and approved the submitted version.

## Conflict of Interest

The authors declare that the research was conducted in the absence of any commercial or financial relationships that could be construed as a potential conflict of interest.

## References

[B1] Al-Younis I. Wong A. Lemtiri-Chlieh F. Schmöckel S. Tester M. Gehring C. . (2018). The *Arabidopsis thaliana* K+-Uptake Permease 5 (AtKUP5) contains a functional cytosolic adenylate cyclase essential for K+ transport. Front. Plant Sci. 9:1645. 10.3389/fpls.2018.01645 30483296 PMC6243130

[B2] Angkawijaya A. E. Nguyen V. C. Gunawan F. Nakamura Y. (2020). A pair of Arabidopsis diacylglycerol kinases essential for gametogenesis and endoplasmic reticulum phospholipid metabolism in leaves and flowers. Plant Cell 32, 2602–2620. 10.1105/tpc.20.00251 32471859 PMC7401011

[B3] Bianchet C. Wong A. Quaglia M. Alqurashi M. Gehring C. Ntoukakis V. . (2019). An *Arabidopsis thaliana* leucine-rich repeat protein harbors an adenylyl cyclase catalytic center and affects responses to pathogens. J. Plant Physiol. 232, 12–22. 10.1016/j.jplph.2018.10.025 30530199

[B4] Bonetta R. Valentino G. (2020). Machine learning techniques for protein function prediction. Proteins 88, 397–413. 10.1002/prot.25832 31603244

[B5] Bowler C. Neuhaus G. Yamagata H. Chua N. H. (1994). Cyclic GMP and calcium mediate phytochrome phototransduction. Cell 77, 73–81. 10.1016/0092-8674(94)90236-4 8156599

[B6] Brady S. M. Orlando D. A. Lee J.-Y. Wang J. Y. Koch J. Dinneny J. R. . (2007). A high-resolution root spatiotemporal map reveals dominant expression patterns. Science 318, 801–806. 10.1126/science.1146265 17975066

[B7] Cai Y. Wang J. Deng L. (2020). SDN2GO: an integrated deep learning model for protein function prediction. Front. Bioeng. Biotechnol. 8:391. 10.3389/fbioe.2020.00391 32411695 PMC7201018

[B8] Chatukuta P. Dikobe T. B. Kawadza D. T. Sehlabane K. S. Takundwa M. M. Wong A. . (2018). An Arabidopsis clathrin assembly protein with a predicted role in plant defense can function as an adenylate cyclase. Biomolecules 8:15. 10.3390/biom8020015 29570675 PMC6022867

[B9] Domingos P. Prado A. M. Wong A. Gehring C. Feijo J. A. (2015). Nitric oxide: a multitasked signaling gas in plants. Mol. Plant 8, 506–520. 10.1016/j.molp.2014.12.010 25680232

[B10] Donaldson L. Ludidi N. Knight M. R. Gehring C. Denby K. (2004). Salt and osmotic stress cause rapid increases in *Arabidopsis thaliana* cGMP levels. FEBS. Lett. 569, 317–320. 10.1016/j.febslet.2004.06.016 15225654

[B11] Durner J. Wendehenne D. Klessig D. F. (1998). Defense gene induction in tobacco by nitric oxide, cyclic GMP, and cyclic ADP-ribose. Proc. Natl. Acad. Sci. U.S.A. 95, 10328–10333. 10.1073/pnas.95.17.10328 9707647 PMC21508

[B12] Ederli L. Meier S. Borgogni A. Reale L. Ferranti F. Gehring C. . (2009). Ozone and nitric oxide induce cGMP-dependent and -independent transcription of defence genes in tobacco. New. Phytol. 181, 860–870. 10.1111/j.1469-8137.2008.02711.x 19140946

[B13] Feijo J. A. Costa S. S. Prado A. M. Becker J. D. Certal A. C. (2004). Signalling by tips. Curr. Opin. Plant.Biol. 7, 589–598. 10.1016/j.pbi.2004.07.014 15337103

[B14] Freihat L. Muleya V. Manallack D. T. Wheeler J. I. Irving H. R. (2014). Comparison of moonlighting guanylate cyclases: roles in signal direction? Biochem. Soc. Trans. 42, 1773–1779. 10.1042/BST20140223 25399605

[B15] Freihat L. A. Wheeler J. I. Wong A. Turek I. Manallack D. T. Irving H. R. (2019). IRAK3 modulates downstream innate immune signalling through its guanylate cyclase activity. Sci. Rep. 9:15468. 10.1038/s41598-019-51913-3 31664109 PMC6820782

[B16] Gattiker A. Gasteiger E. Bairoch A. (2002). ScanProsite: a reference implementation of a PROSITE scanning tool. Appl. Bioinformatics 1, 107–108. 15130850

[B17] Gehring C. (2010). Adenyl cyclases and cAMP in plant signaling - past and present. Cell Commun. Signal. 8:15. 10.1186/1478-811X-8-15 20579354 PMC2907374

[B18] Gehring C. Turek I. S. (2017). Cyclic nucleotide monophosphates and their cyclases in plant signaling. Front. Plant Sci. 8:1704. 10.3389/fpls.2017.01704 29046682 PMC5632652

[B19] Guo T. Fang Y. (2014). Functional organization and dynamics of the cell nucleus. Front. Plant Sci. 5:378. 10.3389/fpls.2014.00378 25161658 PMC4130368

[B20] Hartwig C. Bahre H. Wolter S. Beckert U. Kaever V. Seifert R. (2014). cAMP, cGMP, cCMP and cUMP concentrations across the tree of life: High cCMP and cUMP levels in astrocytes. Neurosci. Lett. 579, 183–187. 10.1016/j.neulet.2014.07.019 25062586

[B21] Hussain J. Chen J. Locato V. Sabetta W. Behera S. Cimini S. . (2016). Constitutive cyclic GMP accumulation in *Arabidopsis thaliana* compromises systemic acquired resistance induced by an avirulent pathogen by modulating local signals. Sci. Rep. 6:36423. 10.1038/srep36423 27811978 PMC5095659

[B22] Irving H. R. Cahill D. M. Gehring C. (2018). Moonlighting Proteins and Their Role in the Control of Signaling Microenvironments, as Exemplified by cGMP and Phytosulfokine Receptor 1 (PSKR1). Front. Plant Sci. 9:415. 10.3389/fpls.2018.00415 29643865 PMC5883070

[B23] Irving H. R. Kwezi L. Wheeler J. Gehring C. (2012). Moonlighting kinases with guanylate cyclase activity can tune regulatory signal networks. Plant Signal. Behav. 7:201–204. 10.4161/psb.18891 22353864 PMC3405710

[B24] Isner J. C. Maathuis F. J. (2011). Measurement of cellular cGMP in plant cells and tissues using the endogenous fluorescent reporter FlincG. Plant J. 65:329–334. 10.1111/j.1365-313X.2010.04418.x 21223396

[B25] Isner J. C. Nuhse T. Maathuis F. J. (2012). The cyclic nucleotide cGMP is involved in plant hormone signalling and alters phosphorylation of *Arabidopsis thaliana* root proteins. J. Exp. Bot. 63, 3199–3205. 10.1093/jxb/ers045 22345640 PMC3350932

[B26] Jeffery C. J. (1999). Moonlighting proteins. Trends Biochem. Sci. 24, 8–11. 10.1016/S0968-0004(98)01335-8 10087914

[B27] Joudoi T. Shichiri Y. Kamizono N. Akaike T. Sawa T. Yoshitake J. . (2013). Nitrated cyclic GMP modulates guard cell signaling in Arabidopsis. Plant Cell 25, 558–571. 10.1105/tpc.112.105049 23396828 PMC3608778

[B28] Kwezi L. Meier S. Mungur L. Ruzvidzo O. Irving H. Gehring C. (2007). The *Arabidopsis thaliana* brassinosteroid receptor (AtBRI1) contains a domain that functions as a guanylyl cyclase *in vitro*. PLoS ONE 2:e449. 10.1371/journal.pone.0000449 17520012 PMC1867859

[B29] Kwiatkowski M. Wong A. Kozakiewicz A. Gehring C. Jaworski K. (2021). A tandem motif-based and structural approach can identify hidden functional phosphodiesterases. Comput, Struct. Biotechnol. J. 19, 970–975. 10.1016/j.csbj.2021.01.036 33613864 PMC7873575

[B30] Lee D. Redfern O. Orengo C. (2007). Predicting protein function from sequence and structure. Nat. Rev. Mol. Cell Biol. 8, 995–1005. 10.1038/nrm2281 18037900

[B31] Lee K. P. Liu K. Kim E. Y. Medina-Puche L. Dong H. Duan J. . (2020). Plant natriuretic peptide A and its putative receptor PNP-R2 antagonize salicylic acid-mediated signaling and cell death. Plant Cell 32, 2237–2250. 10.1105/tpc.20.00018 32409317 PMC7346577

[B32] Levskaya A. Weiner O. D. Lim W. A. Voigt C. A. (2009). Spatiotemporal control of cell signalling using a light-switchable protein interaction. Nature 461, 997–1001. 10.1038/nature08446 19749742 PMC2989900

[B33] Littmann M. Heinzinger M. Dallago C. Olenyi T. Rost B. (2021). Embeddings from deep learning transfer GO annotations beyond homology. Sci. Rep. 11:1160. 10.1038/s41598-020-80786-0 33441905 PMC7806674

[B34] Loewenstein Y. Raimondo D. Redfern O. C. Watson J. Frishman D. Linial M. . (2009). Protein function annotation by homology-based inference. Genome Biol. 10, 207. 10.1186/gb-2009-10-2-207 19226439 PMC2688287

[B35] Ludidi N. Gehring C. (2003). Identification of a novel protein with guanylyl cyclase activity in *Arabidopsis thaliana*. J. Biol. Chem. 278, 6490–6494. 10.1074/jbc.M210983200 12482758

[B36] Maathuis F. J. Sanders D. (2001). Sodium uptake in Arabidopsis roots is regulated by cyclic nucleotides. Plant Physiol. 127, 1617–1625. 10.1104/pp.010502 11743106 PMC133566

[B37] Mahlich Y. Steinegger M. Rost B. Bromberg Y. (2018). HFSP: high speed homology-driven function annotation of proteins. Bioinformatics 34, i304–i312. 10.1093/bioinformatics/bty262 29950013 PMC6022561

[B38] Malukani K. K. Ranjan A. Hota S. J. Patel H. K. Sonti R. V. (2020). Dual activities of receptor-like kinase OsWAKL21.2 induce immune responses. Plant Physiol. 183, 1345–1363. 10.1104/pp.19.01579 32354878 PMC7333719

[B39] Marchler-Bauer A. Lu S. Anderson J. B. Chitsaz F. Derbyshire M. K. DeWeese-Scott C. . (2011). CDD: a Conserved Domain Database for the functional annotation of proteins. Nucleic Acids Res. 39(Database issue), D225–D229. 10.1093/nar/gkq1189 21109532 PMC3013737

[B40] Marondedze C. Groen A. J. Thomas L. Lilley K. S. Gehring C. (2016). A quantitative phosphoproteome analysis of cGMP-dependent cellular responses in Arabidopsis thaliana. Mol. Plant 9:621–623. 10.1016/j.molp.2015.11.007 26658240

[B41] Martinez-Atienza J. Van Ingelgem C. Roef L. Maathuis F. J. (2007). Plant cyclic nucleotide signalling: facts and fiction. Plant Signal. Behav. 2, 540–543. 10.4161/psb.2.6.4789 19704553 PMC2634363

[B42] McGinnis S. Madden T. L. (2004). BLAST: at the core of a powerful and diverse set of sequence analysis tools. Nucleic Acids Res. 32, W20–25. 10.1093/nar/gkh435 15215342 PMC441573

[B43] McInnis S. M. Desikan R. Hancock J. T. Hiscock S. J. (2006). Production of reactive oxygen species and reactive nitrogen species by angiosperm stigmas and pollen: Potential signalling crosstalk? New Phytol. 172, 221–228. 10.1111/j.1469-8137.2006.01875.x 16995910

[B44] Melcher K. Ng L. M. Zhou X. E. Soon F. F. Xu Y. Suino-Powell K. M. . (2009). A gate-latch-lock mechanism for hormone signalling by abscisic acid receptors. Nature 462, 602–608. 10.1038/nature08613 19898420 PMC2810868

[B45] Morris G. M. Huey R. Lindstrom W. Sanner M. F. Belew R. K. Goodsell D. S. . (2009). Autodock4 and AutoDockTools4: automated docking with selective receptor flexibility. J. Comput. Chem. 16, 2785–2791. 10.1002/jcc.21256 19399780 PMC2760638

[B46] Mulaudzi T. Ludidi N. Ruzvidzo O. Morse M. Hendricks N. Iwuoha E. . (2011). Identification of a novel *Arabidopsis thaliana* nitric oxide-binding molecule with guanylate cyclase activity *in vitro*. FEBS Lett. 585, 2693–2697. 10.1016/j.febslet.2011.07.023 21820435

[B47] Muleya V. Wheeler J. I. Ruzvidzo O. Freihat L. Manallack D. T. Gehring C. . (2014). Calcium is the switch in the moonlighting dual function of the ligand-activated receptor kinase phytosulfokine receptor 1. Cell Commun. Signal. 12:60. 10.1186/s12964-014-0060-z 25245092 PMC4180545

[B48] Neuhaus G. Bowler C. Hiratsuka K. Yamagata H. Chua N. H. (1997). Phytochrome-regulated repression of gene expression requires calcium and cGMP. EMBO J. 16, 2554–2564. 10.1093/emboj/16.10.2554 9184203 PMC1169867

[B49] Olea C. Herzik M. A. Kuriyan J. Marletta M. A. (2010). Structural insights into the molecular mechanism of H-NOX activation. Protein Sci. 19, 881–887. 10.1002/pro.357 20162612 PMC2867026

[B50] Ooi A. Lemtiri-Chlieh F. Wong A. Gehring C. (2017). Direct modulation of the guard cell outward-rectifying potassium channel (GORK) by abscisic acid. Mol. Plant 10, 1469–1472. 10.1016/j.molp.2017.08.010 28844521

[B51] Park S. Y. Fung P. Nishimura N. Jensen D. R. Fujii H. Zhao Y. . (2009). Abscisic acid inhibits type 2C protein phosphatases via the PYR/PYL family of START proteins. Science 324, 1068–1071. 10.1126/science.1173041 19407142 PMC2827199

[B52] Pasqualini S. Cresti M. Del Casino C. Faleri C. Frenguelli G. Tedeschini E. . (2015). Roles for NO and ROS signalling in pollen germination and pollen-tube elongation in Cupressus arizonica. Biol. Plant. 59, 735–744. 10.1007/s10535-015-0538-6

[B53] Pasqualini S. Tedeschini E. Frenguelli G. Wopfner N. Ferreira F. D'Amato G. . (2011). Ozone affects pollen viability and NAD(P)H oxidase release from Ambrosia artemisiifolia pollen. Environ Pollut. 159:2823–30. 10.1016/j.envpol.2011.05.003 21605929 PMC3173721

[B54] Pellicena P. Karow D. S. Boon E. M. Marletta M. A. Kuriyan J. (2004). Crystal structure of an oxygen-binding heme domain related to soluble guanylate cyclases. Proc. Natl. Acad. Sci. U.S.A. 101, 12854–12859. 10.1073/pnas.0405188101 15326296 PMC516465

[B55] Pettersen E. F. Goddard T. D. Huang C. C. Couch G. S. Greenblatt D. M. Meng E. C. . (2004). UCSF chimera–a visualization system for exploratory research and analysis. J. Comput. Chem. 25, 1605–1612. 10.1002/jcc.20084 15264254

[B56] Prado A. M. Colaço R. Moreno N. Silva A. C. Feijó J. A. (2008). Targeting of pollen tubes to ovules is dependent on nitric oxide (NO) signaling. Mol. Plant 1, 703–714. 10.1093/mp/ssn034 19825574

[B57] Prado A. M. Porterfield D. M. Feijó J. A. (2004). Nitric oxide is involved in growth regulation and re-orientation of pollen tubes. Development 131, 2707–2714. 10.1242/dev.01153 15128654

[B58] Rahman H. Wang X. Y. Xu Y. P. He Y.-H. Cai X.-Z. (2020). Characterization of tomato protein kinases embedding guanylate cyclase catalytic center motif. Sci. Rep. 10:4078. 10.1038/s41598-020-61000-7 32139792 PMC7057975

[B59] Rifaioglu A. S. Dogan T. Martin M. J. Cetin-Atalay R. Atalay V. (2019). DEEPred: automated protein function prediction with multi-task feed-forward deep neural networks. Sci. Rep. 9:7344. 10.1038/s41598-019-43708-3 31089211 PMC6517386

[B60] Ruzvidzo O. Gehring C. Wong A. (2019). New perspectives on plant adenylyl cyclases. Front. Mol. Biosci. 6:136. 10.3389/fmolb.2019.00136 31850369 PMC6901789

[B61] Sali A. Blundell T. L. (1993). Comparative protein modelling by satisfaction of spatial restraints. J. Mol. Biol. 234, 779–815. 10.1006/jmbi.1993.1626 8254673

[B62] Sanner M. F. (1999). Python: a programming language for software integration and development. J. Mol. Graphics Mod. 17, 57–61. 10660911

[B63] Shen Q. Zhan X. Yang P. Li J. Chen J. Tang B. . (2019). Dual activities of plant cGMP-dependent protein kinase and its roles in gibberellin signaling and salt stress. Plant Cell 31, 3073–3091. 10.1105/tpc.19.00510 31575723 PMC6925016

[B64] Sigrist C. J. de Castro E. Cerutti L. Cuche B. A. Hulo N. Bridge A. . (2013). New and continuing developments at PROSITE. Nucleic Acids Res. 41(Database issue), D344–D347. 10.1093/nar/gks1067 23161676 PMC3531220

[B65] Su B. Qian Z. Li T. Zhou Y. Wong A. (2019). PlantMP: a database for moonlighting plant proteins. Database 2019:baz050. 10.1093/database/baz050 31032837 PMC6482322

[B66] Swiezawska B. Duszyn M. Jaworski K. Szmidt-Jaworska A. (2018). Downstream targets of cyclic nucleotides in plants. Front. Plant Sci. 9:1428. 10.3389/fpls.2018.01428 30327660 PMC6174285

[B67] Swiezawska B. Duszyn M. Kwiatkowski M. Jaworski K. Pawełek A. Szmidt-Jaworska A. (2020). Brachypodium distachyon triphosphate tunnel metalloenzyme 3 is both a triphosphatase and an adenylyl cyclase upregulated by mechanical wounding. FEBS Lett. 594, 1101–1111. 10.1002/1873-3468.13701 31785160

[B68] Swiezawska B. Jaworski K. Duszyn M. Pawełek A. Szmidt-Jaworska A. (2017). The Hippeastrum hybridum PepR1 gene (HpPepR1) encodes a functional guanylyl cyclase and is involved in early response to fungal infection. J. Plant Physiol. 216, 100–107. 10.1016/j.jplph.2017.05.024 28609666

[B69] Swiezawska B. Jaworski K. Szewczuk P. Pawełek A. Szmidt-Jaworska A. (2015). Identification of a *Hippeastrum hybridum* guanylyl cyclase responsive to wounding and pathogen infection. J. Plant Physiol. 189, 77–86. 10.1016/j.jplph.2015.09.014 26523507

[B70] Swiezawska-Boniecka B. Duszyn M. Kwiatkowski M. Szmidt-Jaworska A. Jaworski K. (2021). Cross talk between cyclic nucleotides and calcium signaling pathways in plants-achievements and prospects. Front. Plant Sci. 12:643560. 10.3389/fpls.2021.643560 33664763 PMC7921789

[B71] Szmidt-Jaworska A. Jaworski K. Pawelek A. Kopcewicz J. (2009). Molecular cloning and characterization of a guanylyl cyclase, PNGC-1, involved in light signaling in Pharbitis nil. J. Plant Growth Regul. 28, 367–380. 10.1007/s00344-009-9105-8

[B72] Trott O. Olson A. J. (2010). AutoDock Vina: Improving the speed and accuracy of docking with a new scoring function, efficient optimization, and multithreading. J. Comput. Chem. 31, 455–461. 10.1002/jcc.21334 19499576 PMC3041641

[B73] Turek I. Gehring C. (2016). The plant natriuretic peptide receptor is a guanylyl cyclase and enables cGMP-dependent signaling. Plant Mol. Biol. 91, 275–286. 10.1007/s11103-016-0465-8 26945740

[B74] Turek I. Irving H. (2021). Moonlighting proteins shine new light on molecular signaling niches. Int. J. Mol. Sci. 22:1367. 10.3390/ijms22031367 33573037 PMC7866414

[B75] Turek I. Wheeler J. Bartels S. Szczurek J. Wang Y. H. Taylor P. . (2020). A natriuretic peptide from *Arabidopsis thaliana* (AtPNP-A) can modulate catalase 2 activity. Sci. Rep. 10:19632. 10.1038/s41598-020-76676-0 33184368 PMC7665192

[B76] Vaz Dias F. Serrazina S. Vitorino M. Marchese D. Heilmann I. Godinho M. . (2019). A role for diacylglycerol kinase 4 in signalling crosstalk during Arabidopsis pollen tube growth. New Phytol. 222, 1434–1446. 10.1111/nph.15674 30628082

[B77] Wang Y. Chen T. Zhang C. Hao H. Liu P. Zheng M. . (2009). Nitric oxide modulates the influx of extracellular Ca2+ and actin filament organization during cell wall construction in Pinus bungeana pollen tubes. New Phytol. 182, 851–862. 10.1111/j.1469-8137.2009.02820.x 19646068

[B78] Wang Y.-H. Li X.-C. Zhu-Ge Q. Jiang X. Wang W. D. Fang W. P. . (2012). Nitric oxide participates in cold-inhibited Camellia sinensis pollen germination and tube growth partly via cGMP *in vitro*. PLoS ONE 7:e52436. 10.1371/journal.pone.0052436 23272244 PMC3525538

[B79] Wheeler J. I. Wong A. Marondedze C. Groen A. J. Kwezi L. Freihat L. . (2017). The brassinosteroid receptor BRI1 can generate cGMP enabling cGMP-dependent downstream signaling. Plant J. 91, 590–600. 10.1111/tpj.13589 28482142

[B80] Wong A. Donaldson L. Portes M. T. Eppinger J. Feijó J. A. Gehring C. (2020). Arabidopsis Diacylglycerol Kinase4 is involved in nitric oxide-dependent pollen tube guidance and fertilization. Development 147:dev183715. 10.1242/dev.183715 32220864 PMC13110872

[B81] Wong A. Gehring C. (2013a). Computational identification of candidate nucleotide cyclases in higher plants. Methods Mol. Biol. 1016, 195–205. 10.1007/978-1-62703-441-8_13 23681580

[B82] Wong A. Gehring C. (2013b). The *Arabidopsis thaliana* proteome harbors undiscovered multi-domain molecules with functional guanylyl cyclase catalytic centers. Cell Commun. Signal. 11:48. 10.1186/1478-811X-11-48 23835195 PMC3726316

[B83] Wong A. Gehring C. Irving H. R. (2015). Conserved functional motifs and homology modelling to predict hidden moonlighting functional sites. Front. Bioeng. Biotechnol. 3:82. 10.3389/fbioe.2015.00082 26106597 PMC4460814

[B84] Wong A. Tian X. Gehring C. Marondedze C. (2018). Discovery of novel functional centers with rationally designed amino acid motifs. Comput. Struct. Biotechnol. J. 16, 70–76. 10.1016/j.csbj.2018.02.007 29977479 PMC6026216

[B85] Wong A. Tian X. Yang Y. Gehring C. (2021). Identification of potential nitric oxide sensing proteins using the H-NOX motif. Mol. Plant 14, 195–197. 10.1016/j.molp.2020.11.015 33249236

[B86] Wu C. H. Huang H. Yeh L. S. Barker W. C. (2003). Protein family classification and functional annotation. Comput. Biol. Chem. 27, 37–47. 10.1016/S1476-9271(02)00098-1 12798038

[B87] Xu N. Fu D. Li S. Wang Y. Wong A. (2018a). GCPred: a web tool for guanylyl cyclase functional centre prediction from amino acid sequence. Bioinformatics 34, 2134–2135. 10.1093/bioinformatics/bty067 29420675

[B88] Xu N. Zhang C. Lim L. L. Wong A. (2018b). Bioinformatic analysis of nucleotide cyclase functional centers and development of ACPred webserver, in Proceedings of the 2018 ACM International Conference on Bioinformatics, Computational Biology, and Health Informatics (BCB '18). (New York, NY: Association for Computing Machinery), 122–129. 10.1145/3233547.3233549

[B89] Yan T. Yoo D. Berardini T. Z. Mueller L. A. Weems D. C. Weng S. . (2005). PatMatch: a program for finding patterns in peptide and nucleotide sequences. Nucleic Acids Res. 33, W262–W266. 10.1093/nar/gki368 15980466 PMC1160129

[B90] Zarban R. Vogler M. Wong A. Eppinger J. Al-Babili S. Gehring C. (2019). Discovery of a nitric oxide-responsive protein in *Arabidopsis thaliana*. Molecules 24, 2691. 10.3390/molecules24152691 31344907 PMC6696476

[B91] Zhang L. Ma H. (2012). Complex evolutionary history and diverse domain organization of SET proteins suggest divergent regulatory interactions. New Phytol. 195, 248–263. 10.1111/j.1469-8137.2012.04143.x 22510098

