# Supplementary material for: Computational Identification of Functional Centers in Complex Proteins: A Step-by-Step Guide With Examples
Source: Front Bioinform. 2021 Mar 25;1:652286. doi: 10.3389/fbinf.2021.652286 (PMC9581015; doi:10.3389/fbinf.2021.652286)
Supplement: Supplementary file 1 [file Data_Sheet_1.PDF]

Supplementary Information

Title

Computational identification of functional centers in complex proteins: A step-by-step guide with examples

Authors

Wei Zhou[1, #], Wei Chi[1, #], Wanting Shen[1], Wanying Dou[2], Junyi Wang[1], Xuechen Tian[1], Christoph Gehring[3] and Aloysius Wong[1, 4, *]

Affiliations

[1] Department of Biology, College of Science and Technology, Wenzhou-Kean University, 88 Daxue Road, Ouhai, Wenzhou, Zhejiang Province 325060, China

[2] Department of Computer Science, College of Science and Technology, Wenzhou-Kean University, 88 Daxue Road, Ouhai, Wenzhou, Zhejiang Province 325060, China

[3] Department of Chemistry, Biology & Biotechnology, University of Perugia, I-06121 Perugia, Italy

[4] Zhejiang Bioinformatics International Science and Technology Cooperation Center of Wenzhou-Kean University

[#] These authors contributed equally to this work

* To whom correspondence should be addressed: Aloysius Wong (alwong@kean.edu)

Supplementary Figure S1

```
          1                                                               65
Tt        MKGTIVGTWIKTLRDLYGNDVVDESLKSVGWEPDRVITPLEDIDDDEVRRIFAKVSEKTGKNVNE
Sw        MMGMVFTGLMELIEDEFGYETLDTLLESCELQSEGIYTSVGSYDHQELLQLVVKLSEVSSVPVTE
Pa        MKGIIFVKLNQFVDELWGDEFWDELLQDAELPSDGIYTSVATYDDAELFTLVGLIMGKKGLTGQQ
Np        MYGLVNKAIQDMVCSRFGEETWKQIKHKAEV-DVDVFLSMEGYPDDITHKLVKAASVILSLSPKQ
Lp        MKGIIFNEFLNFVEKSESYTLVDQIIMDSHLKSNGAYTSIGTYAPEELFQLVKALAMKNGKPTSM
Vf        MKGIIFSEFLELVEDKFGLEVCQQML-DENN-DEGAYTAVGTYDHKHLVKLIISLSKVTGVSIED
Ce        MYGLIIDHIATYIKEKYGESTWSEVKFVSGVTDDTFQMDKKFSEGLS-HKLIWACHDVTGDPVDE
Dm        MYGFVNYALELLVLKHFGEEIWEKIKKKAMVSMEGQFLVRQIYDDEITYNLIGAAVEILNIPADD
Rn        MYGFVNHALELLVIRNYGPEVWEDIKKEAQLDEEGQFLVRIIYDDSKTYDLVAAASKVLNLNAGE
Hm        MYGFVNHALELLVIRNYGPEVWEDIKKEAQLDEEGQFLVRIIYDDSKTYDLVAAASKVLNLNAGE
AtDGK4    EIVDPPYSLKATQECYIDQNLEIEGEIPPSTNGYEGVFYNYFSIGMDAQVAYGFHHLRNEKPYLA
AtLRB3    GLEIQSEDVVYEVVLKWVKSHYSVLEARQEVLGSHLARYIRFPHMTTDRLKKILTSNDFRPSVAS
AtNOGC1   ------------------------------------------------------------MVPAV

          66                                                             130
Tt        IWREVGRQNIKTFSEWFPSYFAGR---RLVNFLMMMDEVHLQLTKMIKGATPPRLIAKPVAKDA-
Sw        LVRLFGKKLFVELIEGHPEIANEMKDSFDLLSKIDSF-IHVEVYKLYPQAELPKFTCDRLGDND-
Pa        AQMAFGQWMFKQLLEAAPPEAHKFTDVFSFLYGVQDV-IHVEVKKLNPEAILPEFEFIEETENS-
Np        IMQAFGEFWVQYTAQEGYGEMLDMSGDTLPEFLENLDNLHARVGVSFPKLQPPSFECTDMEENS-
Lp        ILQEYGEYLFEVFAKKYPQFFREKKSVFQFLEALETH-IHFGVKKLYDHTELPHFECIQYHSQKQ
Vf        LQQVYGKSVFLTLFQSMPELDGQALNTFEFIKQVESY-IHLEVKKLYAEANPPRFKFISSTETE-
Ce        LMTNIGTSFYKFLTKFEFNKVLRVLGRTFPQFLNGLDNLHEYLRFTFPKLKPPSFYCEHESRTG-
Dm        ILELFGKTFFEFCQDSGYDKILQVLGATPRDFLQNLDALHDHLGTLYPGMRAPSFRCTEKDGE--
Rn        ILQMFGKMFFVFCQESGYDTILRVLGSNVREFLQNLDALHDHLATIYPGMRAPSFRCTDAEKGKG
Hm        ILQMFGKMFFVFCQESGYDTILRVLGSNVREFLQNLDALHDHLATIYPGMRAPSFRCTDAEKGKG
AtDGK4    NGPIANKIIYSGYGCSQGWFLTHCINDPGLRGLKNIMTLHIKKLDSSEWEKVPVPKSVRAVVALN
AtLRB3    KLVVEALFFKTESLAHQHVLLAHEQPASTSRRFAKRAYVHRPIKIVEFAVPRPQCIIYLDLKRKE
AtNOGC1   NPPTTSNHVAVIGAGAAGLVAARELRREGHSVVVFERGNHIGGVWAYTPNVEPDPLSIDPTRPVI

          131                                                  185
Tt        IEMEYVSKRK-MYDYFLGLIEGSSKFF-KEEISVEEVERGEKDGFSRLKVRIKFK
Sw        IRLHYQSKRP-FASFAEGLLDGCAEYF-KEDFTISRTPETQDSETDVIFNITRAP
Pa        LSFHYLSPRK-MCYFCEGIIEGLADHTGQKVAIE-QPECEHDGDARCVIKVTKVG
Np        LSLHYRSDREGLTPMVIGLIKGLGTRF-DTEVHITQTQ-----NRDEGAEHDEFL
Lp        MEMIYTSSRP-LADFAEGLIRGCIKYH-KENMTIVREN-----LPAKTGFKVRFV
Vf        MVMDYISARC-FSHVCFGLILGCAEHF----NEIIDIQ---MTPVLDDGSQVRFT
Ce        LTLHYRSKRRGFLHYVQGQIRNISQELFQTEVVIELLDIEHDLNLEHVIMRLHFN
Dm        LLLHYYSERPGLEHIVIGIVKAVASKLHGVEVEIDIVKRKGEPIDEAEKERAIAR
Rn        LILHYYSEREGLQDIVIGIIKTVAQQIHGTEIDMKVIQ-----QRSEECDHTQFL
Hm        LILHYYSEREGLQDIVIGIIKTVAQQIHGTEIDMKVIQ-----QRNEECDHTQFL
AtDGK4    L-HSYGSGRNPWGNLKQDYLEKRGFVEAQADDGLLEIFGLKQGWHASFVMVELIS
AtLRB3    CESIYPSSRISSQQFTLGGQGFFLSAQCNMDHLCLIHCFGLFIGMQENGSASASV
AtNOGC1   HSSLYSSLRTIIPQECMGFTDFPFSTRLENGSRDPRRHPGHSEVLAYLRDFVREF
```

H-NOX domain

H-NOX center

Sequence alignment of representative H-NOX domains. Tt = *Thermoanaerobacter tengcongensis* (UniProt ID: Q8RBX6), Sw = *Shewanella woodyi* (UniProt ID: B1KIH6), Pa = *Pseudoalteromonas atlantica* (UniProt ID: Q15VN4), Np = *Nostoc punctiforme* (UniProt ID: B2IZ76), Lp = *Legionella pneumophila* (UniProt ID: Q5WTZ5), Vf = *Vibrio fischeri* (UniProt ID: Q5E1F5), Ce = *Caenorhabditis elegans* (UniProt ID: Q86C56), Dm = *Drosophila melanogaster* (UniProt ID: Q24086), Rn = *Rattus norvegicus* (UniProt ID: P20595), Hs = *Homo sapiens* (UniProt ID: Q02153) and At = *Arabidopsis thaliana*. Bolded letters are conserved amino acids that are also experimentally shown to be crucial for heme-binding and stabilization. Gray bar represents the full H-NOX domain (Pfam ID: PF07700) of approximately 185 amino acids, initially identified by (Iyer et al., 2003) which are highly conserved in prokaryotes and eukaryotes but seemingly missing in plants. Red bar represents the heme-binding center of approximately 35 amino acids from which H-NOX motif Hx(12)Px(14,16)YxSxR was derived from. It includes only the amino acids that are crucial for heme-binding.

Supplementary Figure S2

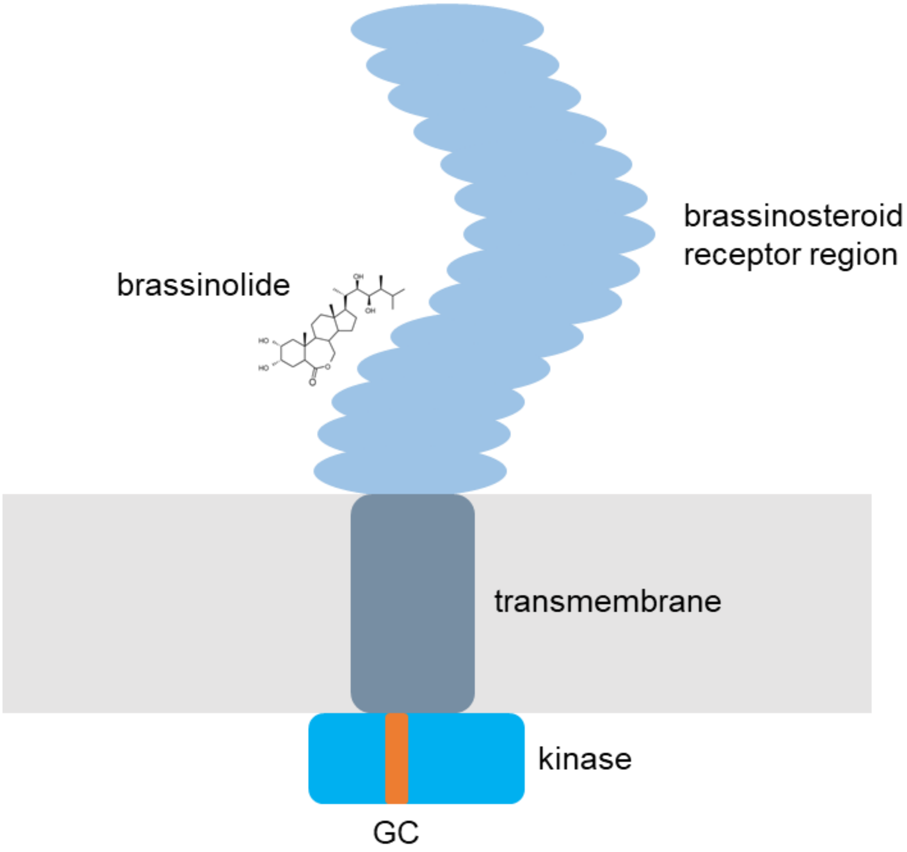

Domain architecture of a GC containing multi-domain protein AtBRI1. The full-length AtBRI1 (UniProt ID: O22476) is 1196 amino acid long and consists of an extracellular brassinosteroid receptor region, a transmembrane, and an intracellular kinase domain. The GC center identified by the 14-amino acid long motif [KS]x[CGS]x(10)[KR], is embedded within the primary kinase domain. The receptor region and kinase domain take up approximately 62.7% and 23.0% of the protein, while the GC center makes up only 0.12% of the entire protein (Wheeler et al., 2017).

Supplementary References

Iyer, L.M., Anantharaman, V. and Aravind, L. (2003). Ancient conserved domains shared by animal soluble guanylyl cyclases and bacterial signaling proteins. *BMC Genomics* 4(1):5.

Wheeler, J. I., Wong, A., Marondedze, C., Groen, A. J., Kwezi, L., Freihat, L., et al. (2017). The brassinosteroid receptor BRI1 can generate cGMP enabling cGMP-dependent downstream signaling. *Plant J.* 91, 590–600.
